# Spatial Patterns of Potentially Hazardous Metals in Soils of Lin’an City, Southeastern China

**DOI:** 10.3390/ijerph16020246

**Published:** 2019-01-16

**Authors:** Shiying Yu, Zhoulun Chen, Keli Zhao, Zhengqian Ye, Luyao Zhang, Jiaqi Dong, Yangfeng Shao, Chaosheng Zhang, Weijun Fu

**Affiliations:** 1 State Key Laboratory of Subtropical Silviculture, Zhejiang A & F University, Lin’an 311300, China; feiyingle@163.com (S.Y.); zhfu021088@126.com (Z.C.); 2 Key Laboratory of Soil Contamination Bioremediation of Zhejiang Province, School of Environmental Sciences and Resources, Zhejiang A & F University, Lin’an 311300, China; yezhq@zafu.edu.cn (Z.Y.); mail_zhangly@163.com (L.Z.); 13735896760@163.com (J.D.); 3 Technology Promotion Center of Agriculture and Forestry in Lin’an City, Hangzhou 311300, China; fuweijun227@163.com; 4 International Network for Environment and Health, School of Geography and Archaeology & Ryan Institute, National University of Ireland, Galway, Ireland; Chaosheng.Zhang@nuigalway.ie

**Keywords:** urban soil, geostatistical analysis, potentially hazardous metals, spatial distribution, ecological risk

## Abstract

Urban soils are strongly related to human health. In this study, Lin’an city was chosen as a typical small-scale city with which to study the spatial variation of potentially hazardous metals (PHMs) in urban soils and their potential ecological risks using multivariate analysis, geostatistics and GIS techniques. A total of 62 soil samples were collected from the study area. The results showed that the average concentrations of total soil Mn, Cu, Zn, Pb, Cr, Cd were 439.42, 42.23, 196.80, 62.55, 63.65, 0.22 mg·kg^−1^, respectively. Compared with the background values and the environmental quality standards, these PHMs were accumulated in urban soils to some extent. The single potential ecological risk indices of PHMs indicated that Pb and Cd had relatively high ecological risks. The pH and most of the PHMs had significant correlations (*p* < 0.05). The principle components analysis (PCA) showed that Pb, Zn and Cu had similar pollution sources related to the vehicles’ exhaust emission; Mn and Cr were mainly from the parent materials; while Cd was from the emission of industrial manufactories. The spatial structures and distributions of PHMs and their corresponding available fractions had strong/moderate spatial autocorrelation, which were influenced by human activities.

## 1. Introduction

Due to economic globalization, human activities continue to increase in the cities, causing the urban population to increase and urban expansion. From 2000 to 2010, urbanization rate in China increased by 15% [1]. However, the rapid process of urbanization is accompanied by unprecedented environmental problems. For example, vehicle exhaust emission, industrial waste disposal and coal combustion led to the enrichment of potentially hazardous metals (PHMs) in urban soils [2]. These PHMs could directly affect the physico-chemical properties of soil and impede the activity of soil microorganisms. The PHMs could also threaten human health by dust inhalation and food ingestion [3]. In addition, urbanization reduces the city wind speed, which is detrimental to the transportation of urban pollutants [4]. Meanwhile, municipal solid waste contains a large amount of abandoned batteries, plastic products, metals, toxic organic compounds and so forth, which can cause serious damage to the soil environment and the growth of crops [5,6,7,8].

In recent years, soils have been used as a diagnostic tool of environmental conditions that influence human health [9,10]. Urban soils are the basic site condition for urban green spaces [11]. Therefore, the research related to urban soil pollution has attracted increasing attention. Manta reported that the vehicle emission was the major source of PHMs pollution in urban soils in Palermo of Italy [12]. Moller et al. [13] found that the discharge of municipal domestic sewage combined with industrial wastewater was an important factor for the increase of PHM contents in urban soils of Damascus, Syria and direct ingestion of soil and inhalation of dust could contribute largely to the accumulation of heavy metal in human and livestock. A study showed that anthropogenic inputs (used oil) could lead to organic pollution of urban soils [14]. In China, much work has been carried out on soils of cities such as Beijing, Guangzhou, Shenyang, Nanjing, Guiyang [15,16,17,18]. However, most of these researches focused on large cities or cities with high-intensity of industrialization. Little information could be found related to PHMs pollution in smaller cities. This is now important as more and more people prefer to live in such cities.

Over the last 35 y, geostatistical techniques such as semivariogram and kriging have been extensively applied to investigate the spatial distribution of continuously varying environmental variables and to incorporate this information into mapping [19,20,21]. Geostatistics has provided an effectively advanced method, which could guarantee high accuracy of the spatial features of soil variables and subsequent spatial interpolation [22,23].

Lin’an, located in southeastern China, is a typical small city in China. In recent years, the normal life of residents have been affected by the rapid development of urbanization [24]. Therefore, detailed information is necessary to provide the basis for urban planning, which could also further be used by other small cities in China. The main objectives of this study were to reveal the spatial variation of PHMs in soils and to identify the PHMs pollution characteristics in the study area using the geostatistics, geographic information system (GIS) technology and multivariate analysis.

## 2. Materials and Methods

### 2.1. Study Area

Lin’an city is located in southeastern China (118°51′~119°52′ E; 29°56′~30°23′ N). It has a subtropical monsoon climate with an average annual temperature of 15.8 °C and an average annual precipitation of 1613.9 mm. The main soil types include yellow soils, red soils, fluvo-aquic soils, paddy soils and saline soils. Lin’an is adjacent to the large city of Hangzhou. It has an urban population of 200,000 and an annual GDP of 95,500 Yuan per person, which is a the moderately developed small-city in Zhejiang Province, China. The main industries of Lin’an include electronics, pharmacy and agricultural products processing. Lin’an is a typical small city in southeastern China.

### 2.2. Soil Sampling and Analysis

**Figure 1:**
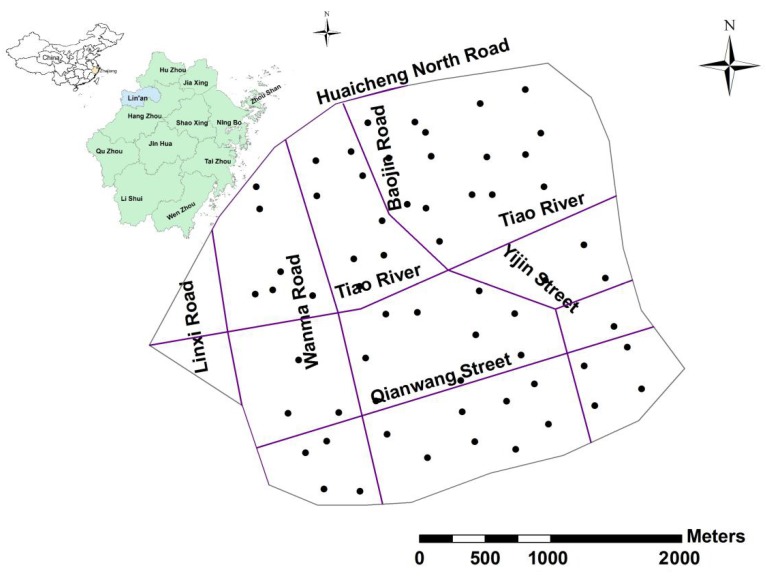
Spatial distribution map of soil sampling sites.

The urban area of Lin’an city was chosen as the study area (Figure 1). A total of 62 top soil samples (0–20 cm) were collected based on a grid sampling scheme of 1 sample per 0.25 km^2^, covering the main urban area of Lin’an city. Among which 20, 15, 12, 8, 7 samples were taken from residential areas, roadsides, public park, schools and agricultural areas, respectively. The sampling location of each sample point was recorded with a portable GPS. The positioning data of GPS was imported into the computer to produce the spatial soil sampling map (Figure 1).

The soil samples were air-dried in laboratory and then sieved through a 2-mm nylon mesh for soil chemical and physical analyses. A portion of the prepared soil samples were ground in an agate mortar to pass through a 0.1 mm pore size sieve and stored in polyethylene bottles.

Soil pH and electrical conductivity (EC) were analyzed with a soil/water ratio of 1:2.5 and 1:5, respectively, in an aqueous suspension [25]. The available concentrations of Mn, Cu, Zn, Pb, Cr, Cd for the soil samples (<2 mm) were extracted using 0.1 mol·L^−1^ HCl. The milled samples (<0.1 mm) of 0.25 g were digested to dryness with the mixture of HF, HNO_3_ and HClO_4_ for measurements of the concentrations of PHMs. Total and available Mn, Cu, Zn, Pb, Cr, Cd concentrations were determined by ICP-OES (Optima 7000 DV, PerkinElmer, Waltham, MA, USA).

For quality assurance of the experiments, the samples were measured in duplicate and the quality control was carried out using the Chinese standardized reference materials (GSS-4 and GSS-15 for soil samples). The analytical quality control showed satisfactory precision throughout.

### 2.3. Pollution Assessment Methods for PHMs

In this study, the single factor pollution index (SFPI) and Nemerow multi-factor pollution index were adopted to evaluate the PHM pollution in urban soils. The formula of single factor contaminant index was as follows [26]:

(1) $$P_{i}=C_{i}/S_{i}$$

Where *P_i_* is the single pollution index of pollutant i; *C_i_* is the measured total concentration of pollutant i; *S_i_* is the background value in soils of Zhejiang Province [27].

The Nemerow multi-factor pollution index takes into account the average and maximum of the SFPI, which could reflects the overall PHMs pollution. The formula was as follows [28]:

(2) $$P_{N}=\sqrt{\frac{1}{2}\left[{\left(\frac{C_{i}}{S_{i}}\right)}_{max}^{2}+{\left(\frac{C_{i}}{S_{i}}\right)}_{ave}^{2}\right]}$$

where *P_N_* is the multiple pollution index; (*C_i_*/*S_i_*)_*max*_ and (*C_i_*/*S_i_*)_*ave*_ represent the maximum and average value of the SFPI, respectively.

According to the Nemerow multi-factor pollution index, the urban soil quality is divided into five levels, including Clean level (*P_N_* ≤ 0.7), Precaution level (0.7 < *P_N_* < 1.0), Slightly polluted level (1.0 < *P_N_* ≤ 2.0), Moderately polluted level (2.0 < *P_N_* ≤ 3.0), Heavily Polluted level (*P_N_* > 3.0).

### 2.4. Potential Ecological Risk Assessment

The potential ecological risk index proposed by Hakanson [29] was used to evaluate PHMs in soils. According to the toxicity of PHMs and their environmental behavior, this method has been widely used by researchers [30,31,32]. The formula is as follows [29]:

(3) $$E_{f}^{i}=T_{f}^{i}\times \frac{C_{i}}{C_{0}}$$

(4) $$RI=\sum_{i=1}^{n}E_{f}^{i}$$

where *C_i_* is the measured concentration of an element in the sample i, *C*_0_ is the soil background value of the element in the study area, $E_{f}^{i}$ is the potential individual ecological risk index for PHM, $T_{f}^{i}$ is the toxicity response factor of PHMs (Pb = Cu = 5; Cr = 2; Zn = Mn = 1; Cd = 30) [29], RI is the comprehensive index of potential ecological risk of the study area. In this study, the soil background value of Zhejiang province was used as the guideline value for calculation.

$E_{f}^{i}$ < 40; *RI* < 150 means low potential ecological risk;

40 ≤ $E_{f}^{i}$ < 80; 150 ≤ *RI* < 300 means moderate potential ecological risk;

80 ≤ $E_{f}^{i}$ < 160; 300 ≤ *RI* < 600 means considerable potential ecological risk;

160 ≤ $E_{f}^{i}$ < 320; *RI* > 600 means very high potential ecological risk;

$E_{f}^{i}$ ≥ 320 means extremely potential ecological risk.

### 2.5. Geostatistical Analysis

Geostatistics is based on the theory of regionalized variables [33]. A semi-variogram is chosen to quantify the spatial variation of an environmental variables and derives essential input parameters for kriging interpolation [34,35]. Geostatistics is widely applied to study environmental variables, which contain both random and structural features in their spatial distributions [36]. In this study, the ordinary kriging interpolation is used to map the spatial distribution and to identify the spatial patterns of studied variables. Ordinary kriging requires that the data should be subject to the normal distribution [37]. Therefore, Kolmogorov-Smirnov (K-S) test combined with kurtosis and skewness values [38,39], are widely applied and considered conservative for normality test. A logarithmic transformation was performed if the raw data did not follow the normal distribution as the highly skewed data can endanger the spatial structure as well as influence the prediction accuracy [21,40].

### 2.6. Data Analysis with Computer Software

The SPSS^®^ for windows (version 22.0) (IBM, Armonk, NY, USA) and Microsoft Excel^®^ statistical software packages were used to calculate descriptive parameters of data and to carry out multi-variates analyses, such as correlation and principal components analyses. Geostatistical analyses were performed with GS+ (v. 7.0) software (Gamma Design, Plainwell, MI, USA). All maps were produced using ArcGIS (v. 10.2) software (ESRI, Redlands, CA, USA).

## 3. Results and Discussion

### 3.1. Descriptive Statistics of the Raw-Data Sets

Descriptive statistics of soil physico-chemical properties and PHMs are shown in Table 1. Soil pH values ranged from 4.64 to 7.33, with an average of 5.66, indicating that the soils were mainly acidic in the study area [41,42]. The mean value of electrical conductivity (EC) was 166.72 μS·cm^−1^, with a wide range of 10.00 to 460.00 μS·cm^−1^, which was related to the variety of soil types in the study area. For example, the high soil EC values were mainly from saline soils. Compared to soil background values of Zhejiang province, all the mean concentrations of total Cu, Zn and Pb were higher than their corresponding background values. Especially for total Pb, its mean value was twice higher than its background value. The average available concentrations of Mn, Cu, Zn, Pb, Cr and Cd were 439.42, 42.23, 196.80, 62.55, 63.65, 0.22 mg·kg^−1^, respectively. Among them, the activation rate of Cd (available fraction/total amount) was the highest, while Cr was the lowest in the soils.

The coefficients of variation (CV) of soil PHMs in study area ranged from 51.29% to 268.51%, which belonged to the medium and strong variation level [43]. The CV values of total Pb, available Pb and available Cd were higher than 100%, which indicated that there was a significant difference between the maximum and minimum values of these two elements. The maximum value of Pb is 40 times higher than its background value in Zhejiang province, indicating that the element Pb was highly enriched in some areas of the study area. Meanwhile, both the kurtosis and skewness values of the soil total Pb and its available fraction were high. Such high positive skewness was likely related to anthropogenic activities [44]. Compared with the large cities of Beijing [16] and Guangzhou [45], some PHMs (Pb, Cu and Cd) in the soils of Lin’an city were at a higher level. Compared with the old industrial city of Xi’an [15], all the PHMs concentrations in the soil of Lin’an city were relatively low. While compared with less developed areas such as Hohhot [46], the PHMs in the study area were high.

### 3.2. Assessment of PHMs Pollution in Soils

In this study, the background values of PHMs in soils of Zhejiang Province were used as evaluation criteria for metal pollution in the soils. The average SFPI values of Pb, Zn, Cu and Cr were higher than 1 (2.05, 1.83, 1.38, 1.14, respectively) (Table 2), among which the Pb had the highest average SFPI, indicating that these metals were enriched in the soils of the study area. The pollution ratio followed the order of Zn > Cu > Pb > Cr > Cd > Mn. The average values of SFPI of Mn and Cd were less than 1 but the concentrations of Mn and Cd in some samples were still higher than the corresponding background values, respectively.

The Nemerow multi-factor index results indicated that no samples belonged to the clean level, while there were 12, 34, 9 and 7 samples belonged to the precaution level, light, moderate and heavy pollution, respectively (Table 3). The corresponding percentages followed the order of light pollution > precaution level > moderate pollution > heavy pollution > clean. About 80% of the soil sampling points were polluted, indicating that the overall pollution status of soils in Lin’an was serious. Some of the samples belong to the heavy pollution level, requiring attention for management.

### 3.3. Ecological Risk Assessment

The average $E_{f}^{i}$ of Mn, Cu, Zn, Pb, Cr and Cd in soils were 0.71, 6.91, 1.83, 10.27, 2.27, 28.73, respectively. They were all less than 40, indicating that these metals posed a low ecological risk. The mean $E_{f}^{i}$ values decreased in the order Cd > Pb > Cu > Cr > Zn > Mn (Table 4). The Pb and Cd posed a higher individual potential ecological risk than the other studied elements in some specific locations such as roadsides. The average *RI* value of the soil in Lin’an was 50.72, indicating that the average pollution degree of PHMs in soils of Lin’an City was relatively low. Ma et al. [47] reported that the urban soils of Changsha city had a moderate potential ecological risk with an average *RI* value of 151.7. The relatively low average *RI* values in Lin’an city may be related to less toxic elements, such as As and low average Cd values.

### 3.4. Correlation and Principal Component Analyses

The correlation between studied variables was analyzed based on Spearman correlation coefficient analysis. The results (Table 5) showed that soil pH was significantly correlated with most of the total PHMs and the available fractions of Mn and Pb, which was consistent with other studies that soil pH was an important factor affecting the activity of metals in soil [43,48]. There was a significant correlation between the total PHMs and their available fractions, among which the total Cd and available Cd had the highest correlation coefficient, indicating that the activity of soil Cd was much stronger than others [48]. Significant correlations between most PHMs were also found, indicating that the sources of PHMs may be similar [5].

The principal component analysis results showed that the first principal component (PC1) explained 50.32% of the total variation, with positive and high loading values of the total Cu, Zn and Pb (>0.8) (Table 6). The strong correlations between the three elements may be related to the same anthropogenic activity [22]. Over the past 50 years, the main source of Pb in urban soils was from vehicle exhaust emission [5,49,50]. Although the use of leaded petrol has been controlled since 2000, the deposited Pb in soils has a long half-life [51]. Therefore, Pb has always been regarded as an important contaminant in urban soils. Copper and Zn were also released into the environment from the emissions of vehicles, tire wear and the loss of automotive components [52,53]. The second principal component (PC2) explained 28.45% of the total variation and showed the high loading values of Mn and Cr (>0.7), indicating that the soil parent materials were their main source [47]. Cadmium alone occupied the third main component, which may be caused by the discharge of “wastes” from electroplating, metallurgy and other industries in urban areas [54].

### 3.5. Spatial Structures of PHMs

The semivariance models and their key parameters are presented in Table 7. The best-fitted theoretical variogram models were based on the mean standardized (MS) and root-mean-square standardized (RMSS) [55,56]. When the MS value is closer to 0 and the RMSS values are closer to 1, the fitted model is optimal [57]. The total Mn, Cd, Pb, Zn, Cr and available Mn, Zn, Cd in soils were all satisfactorily fitted with Gaussian models. Total Cu, available Pb, Cr and pH in soils were well fitted with exponential models, while the available Cu and soil EC were fitted with spherical models. “Nugget/sill” ratio ([C_0_/(C_0_+C)]) is used to measure the strength of the spatial autocorrelation of the studied variables [58]. If the “nugget/sill” ratio is <25%, the variable is considered to have a strong spatial dependence and the spatial variability is mainly caused by natural factors such as soil parent material, topography and climate. A ratio is between 25% and 75% indicates that the variable has moderate spatial dependence and its spatial variability is affected by the combination of soil properties and human activities [21,36]. When the ratio is >75%, the spatial dependence of variable is very weak. The “nugget/sill” ratio of pH, CEC and available Mn, Cu, Zn and Cr in soils ranged from 25% to 75% (Table 7), indicating that random and structural factors were the important factors causing variation. The ratios of other variables were less than 25%, indicating that they were affected by structural factors, whereas the spatial structures of Pb and Cd were strong, which might be related to the influences of long-term anthropogenic activities. Such influences could become part of the structural factors [43]. According to the low “nugget/sill” ratio values, clear spatial patterns of PHMs could be revealed by kriging interpolation [23]. Similar moderate and strong spatial structures of PHMs were also found in Galway city, a small tourist city in Western Ireland [22].

### 3.6. Spatial Distribution Pattern of PHMs and Physicochemical Properties in Soils

**Figure 2:**
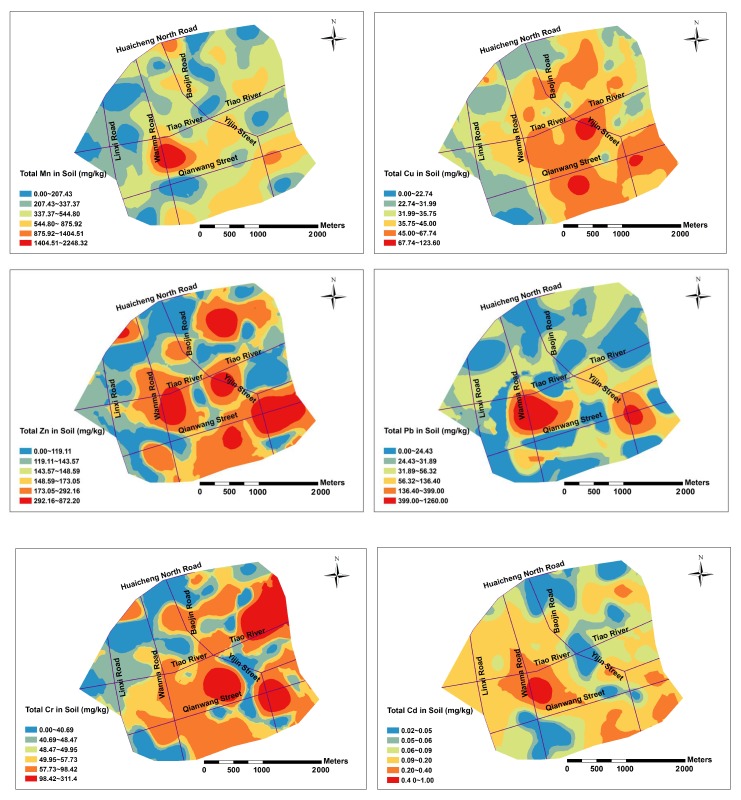
Spatial distribution maps of the total PHMs.

**Figure 3:**
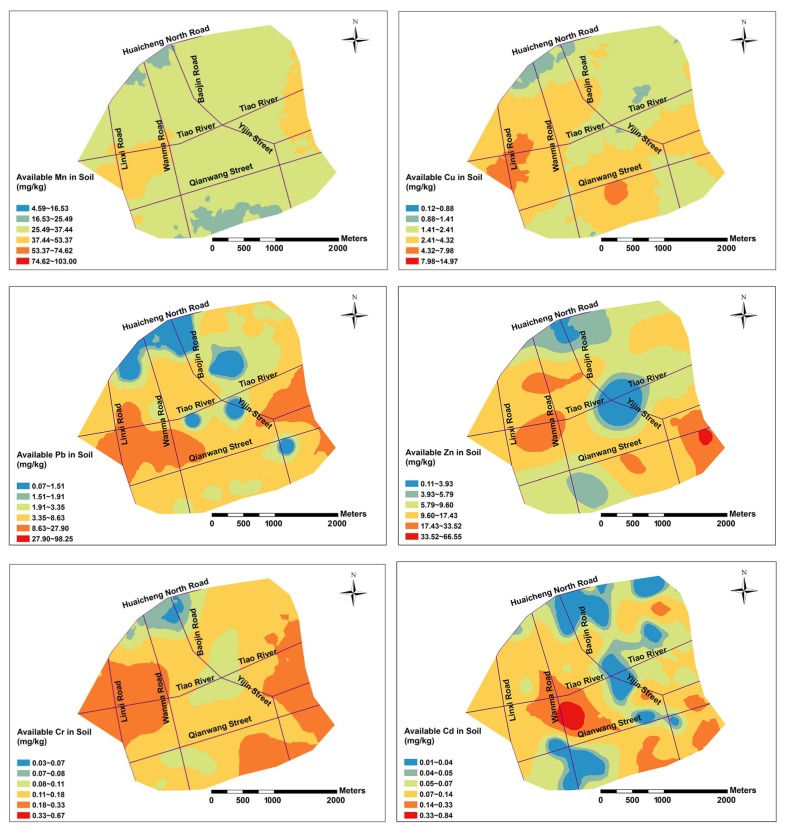

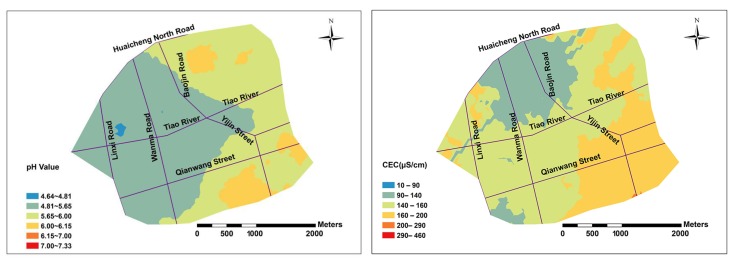
Spatial distribution maps of available fractions of PHMs, pH and CEC.

Figure 2 and Figure 3 describe the spatial distributions of total PHMs, their available fractions, pH and EC based on ordinary Kriging interpolation. The spatial distribution characteristics of total Cu, Zn and Pb in soils had strong similarity. The concentrations of total Cu, Zn and Pb in the central and southeast parts of Lin’an City were high, while they were low in the northwest and southwest of City. The concentrations of total Cr was high in the eastern part of the study area and low in the western part, showing an increasing trend from west to east. Total Cd in soil was high in the central part of the study area, while it was low in the northern part and the southern part, indicating an increasing trend from south to north. The high values of total Cu, Zn and Pb in soils were mainly distributed in Wanma Road and Qianwang Street, which was related to a large number of automobile fitting factories and shops, as well as the heavy traffic and transportation of these two main traffic routes, indicating that transportation and relevant industry were the main factors affecting Cu, Zn and Pb concentrations in soils [22,31].

The pH values were low in the western part of the study area and increased gradually from west to east. Soil CEC showed a decreasing trend from south to north in the study area. The spatial distribution of available Cd in soils was consistent with total Cd in soils, especially the spatial patterns of high values were basically the same, indicating that the available Cd was mainly related to total Cd, while the spatial patterns of available Mn and Cr in soils and total Mn and Cr were quite different, respectively. The concentrations of available Mn was high in the western and eastern parts of the study area and relatively low in the southern and northern parts. The available Cr values were high in the western and southeast parts of the study area and low in the northern part of the study area. The available fractions of PHMs is affected not only by the total amount of corresponding PHMs but also influenced by other factors [59,60].

## 4. Conclusions

The average concentrations of total Mn, Cu, Zn, Pb, Cr and Cd in the soils were 439.42, 42.23, 196.66, 62.55, 63.65 and 0.22 mg·kg^−1^, respectively. Compared with the background values of PHMs in Zhejiang Province, the total Mn, Cu, Zn, Pb, Cr and Cd in soils of Lin’an city were enriched to some extent. There was a significant correlation between soil PHMs metals and pH, indicating that pH had an important effect on the activity and accumulation of PHMs in soils. Soil PHMs in the study area had clear spatial patterns, as the high concentrations of PHMs mainly located in the industrial areas and along traffic routes. The main sources of Cu, Zn and Pb were related to the automobile exhaust emissions, tire wear and the loss of automobile parts, while Cd might be related to the industrial activities. Necessary pollution control measures should be considered with the rapid development of small cities in China.

## Figures and Tables

**Table 1:** Descriptive statistics of physical-chemical properties and PHMs in soils (mg·kg^−1^; EC: µS·cm^−1^).

Variable	Min	Max	Median	Mean	SD	CV/%	Skew	Kurt	Geometric Mean	Background Value ^a^
Mn_Total_	88.16	2248.31	397.33	439.42	305.37	69.49	3.61	20	371.99	555.48
Cu_Tota_	11.73	123.63	37.47	42.23	21.66	51.29	1.73	4.13	37.8	30.54
Zn_Total_	67.49	872.19	158	196.8	142.76	72.54	2.59	8.46	165.73	107.79
Pb_Total_	7.61	1259.62	30.94	62.55	167.95	268.51	6.55	45.27	34.44	30.46
Cr_Total_	18.87	311.37	51.13	63.65	53.89	84.67	3.78	14.61	54.11	55.99
Cd_Total_	0.02	1.12	0.09	0.22	0.15	68.2	5.1	33.1	0.09	0.13
pH	4.64	7.33	5.81	5.66	0.94	16.61	-0.83	0.67	5.57	—
EC	10	460	0.16	166.72	71.83	43.08	1.49	4.63	150.64	—
Mn_avai_	4.59	102.95	34.53	38.41	24.4	63.53	0.83	0.11	30.63	—
Cu_avail._	0.01	14.97	1.89	2.57	2.6	91.17	2.38	7.82	1.49	—
Zn_avail._	0.11	66.55	9.43	13.46	12.81	95.17	1.94	4.4	8.89	—
Pb_avail._	0.07	98.25	2.91	6.01	13.47	224.13	5.83	37.85	2.35	—
Cr_avail._	0.04	0.67	0.16	0.17	0.13	76.47	1.76	3.49	0.14	—
Cd_avail._	0.01	0.84	0.07	0.09	0.11	122.22	5.12	33.47	0.07	—

—: No relevant data, Min: minimum, Max: maximum, SD: standard deviation, CV: coefficient of variation, Skew: skewness, Kurt: kurtosis, Mn_Total_, Cu_Total_, Zn_Total_, Pb_Total_, Cr_Total_, Cd_Total_: total Mn, Cu, Zn, Pb, Cr, Cd in soils, respectively; Mn_avail._, Cu_avail._, Zn_avail._, Pb_avail._, Cr_avail._, Cd_avail._: the availability of Mn, Cu, Zn, Pb, Cr, Cd refers to the acid extractables form in soils, respectively. ^a^ The background value of potentially hazardous metals of soil in the Zhejiang Province [27].

**Table 2:** The evaluated results of single factor pollution index for soil PHMs in Lin’an.

Elements	Background Values as Critical Value in Zhejiang Province			
	Mean	Minimum	Maximum	Pollution Ratio (%)
Mn	0.71	0.14	3.62	16.39
Cu	1.38	0.38	4.05	67.21
Zn	1.83	0.63	8.09	78.69
Pb	2.05	0.25	41.35	52.46
Cr	1.14	0.34	5.56	37.70
Cd	0.96	0.12	8.62	32.79

**Table 3:** Evaluated results of PHMs pollution in soils (%).

Degree of Pollution	Clean *P_N_* < 0.7	Precaution Level 0.7 < *P_N_* < 1	Light Pollution 1 < *P_N_* < 2	Moderate Pollution 2 < *P_N_* < 3	Heavy Pollution *P_N_* > 3
Number of samples	0	12	34	9	7
Percentage (%)	0	20	55	15	11

*P_N_* is Nemerow multi-factor pollution index.

**Table 4:** Ecological Risk index of PHMs in the soils of Lin’an.

Elements	$E_{f}^{i}$	Ratio of Samples with Different Degree of Risk (%)				
		Slight	Moderate	Strong	Very Strong	Extremely Strong
Mn	0.71	100	0	0	0	0
Cu	6.91	100	0	0	0	0
Zn	1.83	100	0	0	0	0
Pb	10.27	98.36	0	1.64	0	0
Cr	2.27	100	0	0	0	0
Cd	28.73	78.69	19.67	0	1.64	0

**Table 5:** Spearman’s correlations between heavy metals in soil.

Variable	Mn_Total_	Cu_Total_	Zn_Total_	Pb_Total_	Cr_Total_	Cr_Total_	pH	CEC	Mn_avail_	Cu_avail_	Zn_avail_	Pb_avail_	Cr_avail_
Cu_Total_	0.47 **												
Zn_Total_	0.48 **	0.75 **											
Pb_Total_	0.34 *	0.85 **	0.68 **										
Cr_Total_	0.18	0.35 **	0.39 **	0.13									
Cd_Total_	0.35 **	0.58 **	0.64 **	049 **	0.10								
pH	0.27 *	0.25 *	0.39 **	0.41 **	−0.15	0.26 *							
CEC	0.06	0.13	0.26 *	0.20	−0.12	0.16	0.31 *						
Mn_avail_	0.26 *	−0.02	0.06	0.12	−0.06	0.45 **	0.26 *	−0.01					
Cu_avail_	−0.04	0.24 *	0.09	0.27 *	0.04	0.33 *	−0.04	0.11	0.38 **				
Zn_avail_	0.35 **	0.26 *	0.29 *	0.46 **	−0.09	0.79 **	0.25	0.25	0.49 **	0.55 **			
Pb_avail_	−0.28 *	−0.12	−0.16	0.28 *	0.01	0.34 **	−0.31 *	−0.11	0.31 *	0.34 **	0.39 **		
Cr_avail_	0.10	0.15	0.09	0.26 *	0.25 *	−0.10	0.20	0.18	0.47 **	0.33 **	0.45 **	0.46 **	
Cd_avail_	0.13	0.12	0.21	0.14	0.10	0.36 **	−0.10	0.10	0.03	0.23	0.24	0.05	0.09

* Significant at 0.05 level; ** Significant at the 0.01 level; Mn_Total_, Cu_Total_, Zn_Total_, Pb_Total_, Cr_Total_, Cd_Total_: total Mn, Cu, Zn, Pb, Cr, Cd in soils, Mn_avail._, Cu_avail._, Zn_avail._, Pb_avail._, Cr_avail._, Cd_avail._: the availability of Mn, Cu, Zn, Pb, Cr, Cd refers to the acid extractables form in soils.

**Table 6:** Matrix of the principal component analysis loadings of soil PHMs.

Elements	Principal Component Loading Factor		
	PC1 (50.32%)	PC2 (28.45%)	PC3 (16.53%)
Mn	0.168	0.782	0.215
Cu	0.895	0.283	0.138
Zn	0.821	0.196	0.241
Pb	0.839	0.251	0.169
Cr	0.287	0.756	0.184
Cd	0.282	0.179	0.786

**Table 7:** The theoretical semivariogram models and the corresponding parameters for PHMs in soils.

Variable	Model	Nugget (*C*_0_)	Sill (*C*_0_ + *C*)	Nugget/Sill [*C*_0_/(*C*_0_ + *C*)]	Range (km)	MS	RMSS
Mn_Total_	Gaussian	124.544	124,668.444	0.10%	0.005	−0.072	1.015
Cd_Total_	Gaussian	0.076	0.684	11.11%	0.004	−0.152	1.039
Cu_Total_	Exponential	75.479	553.778	12.63%	0.006	0.021	1.084
Pb_Total_	Gaussian	42.554	42,596.924	0.10%	0.007	−0.072	1.073
Zn_Total_	Gaussian	238.042	23,034.992	1.03%	0.004	0.010	1.032
Cr_Total_	Gaussian	3.296	3299.030	0.09%	0.004	−0.021	1.093
pH	Exponential	0.018	0.038	47.38%	0.017	−0.005	0.900
CEC	Spherical	0.168	0.278	56.40%	0.038	0.003	0.765
Mn_avail_	Gaussian	0.201	0.588	34.18%	0.011	−0.001	0.739
Cu_avail_	Spherical	5.854	7.496	46.09%	0.006	0.010	0.960
Zn_avail_	Gaussian	0.540	1.275	42.35%	0.007	−0.001	0.939
Pb_avail_	Exponential	0.529	2.540	20.83%	0.006	0.010	0.908
Cd_avail_	Gaussian	0.076	0.684	11.11%	0.004	−0.151	1.039
Cr_avail_	Exponential	0.291	0.592	49.15%	0.012	−0.013	0.880

Mn_Total_, Cu_Total_, Zn_Total_, Pb_Total_, Cr_Total_, Cd_Total_: total Mn, Cu, Zn, Pb, Cr, Cd in soils, Mn_avail._, Cu_avail._, Zn_avail._, Pb_avail._, Cr_avail._, Cd_avail._: the availability of Mn, Cu, Zn, Pb, Cr, Cd refers to the acid extractables form in soils. MS: mean standardized, RMSS: root-mean-square standardized.
